# Two‐stage or not two‐stage? That is the question for IPD meta‐analysis projects

**DOI:** 10.1002/jrsm.1661

**Published:** 2023-08-22

**Authors:** Richard D. Riley, Joie Ensor, Miriam Hattle, Katerina Papadimitropoulou, Tim P. Morris

**Affiliations:** ^1^ Institute of Applied Health Research, College of Medical and Dental Sciences University of Birmingham Birmingham UK; ^2^ School of Medicine Keele University Keele Staffordshire UK; ^3^ Health Economics and Market Access Amaris Consulting Lyon France; ^4^ MRC Clinical Trials Unit at UCL Institute of Clinical Trials and Methodology, UCL London UK

**Keywords:** individual participant data (IPD), meta‐analysis, one‐stage approach, two‐stage approach

## Abstract

Individual participant data meta‐analysis (IPDMA) projects obtain, check, harmonise and synthesise raw data from multiple studies. When undertaking the meta‐analysis, researchers must decide between a two‐stage or a one‐stage approach. In a two‐stage approach, the IPD are first analysed separately within each study to obtain aggregate data (e.g., treatment effect estimates and standard errors); then, in the second stage, these aggregate data are combined in a standard meta‐analysis model (e.g., common‐effect or random‐effects). In a one‐stage approach, the IPD from all studies are analysed in a single step using an appropriate model that accounts for clustering of participants within studies and, potentially, between‐study heterogeneity (e.g., a general or generalised linear mixed model). The best approach to take is debated in the literature, and so here we provide clearer guidance for a broad audience. Both approaches are important tools for IPDMA researchers and neither are a panacea. If most studies in the IPDMA are small (few participants or events), a one‐stage approach is recommended due to using a more exact likelihood. However, in other situations, researchers can choose either approach, carefully following best practice. Some previous claims recommending to always use a one‐stage approach are misleading, and the two‐stage approach will often suffice for most researchers. When differences do arise between the two approaches, often it is caused by researchers using different modelling assumptions or estimation methods, rather than using one or two stages per se.

**Highlights**

**What is already known**

- An individual participant data meta‐analysis (IPDMA) can be undertaken using either a two‐stage or a one‐stage approach.
- In a two‐stage approach, the IPD are first analysed separately within each study to obtain aggregate data; then, in the second stage, these aggregate data are combined in a standard meta‐analysis model.
- In a one‐stage approach, the IPD from all studies are analysed in a single step using an appropriate model that accounts for clustering of participants within studies.
- The best approach to take is debated in the literature.

**What is new**

- If most studies in the IPDMA are small (few participants or events), a one‐stage approach is recommended due to using a more exact likelihood.
- In other situations, researchers can choose either approach, carefully following best practice.
- Previous claims in favour of always using a one‐stage approach are often misleading.
- Neither approach is a panacea, but both are important tools for IPDMA projects.

**Potential impact for *Research Synthesis Methods* readers**

- Unless most studies are small (few participants or events), any observed differences between one‐stage and two‐stage approaches are likely due to researchers using different models, assumptions or estimation methods, rather than using one or two stages per se.
- One‐stage models require great care to specify correctly and so, unless data are sparse, the two‐stage approach will often suffice for most researchers.

## 1 INTRODUCTION

Individual participant data meta‐analysis (IPDMA) projects obtain, check, harmonise and synthesise raw data from multiple studies.^1^ When undertaking an IPDMA, researchers must decide between a two‐stage or a one‐stage approach to data synthesis.^2^, ^3^ For example, in a meta‐analysis of randomised trials comparing a treatment to a control, the two‐stage approach first analyses the IPD separately within each trial to obtain aggregate data, such as the treatment effect estimate and its standard error. The second stage then combines these aggregate data using a standard meta‐analysis model (e.g., inverse‐variance weighting) to produce summary results. In contrast, the one‐stage approach analyses all trials in a single step using an appropriate regression model.^4^ Some methodology articles strongly advocate a one‐stage approach,^5^, ^6^ whilst others defend the two‐stage.^7^, ^8^, ^9^ This article aims to provide clearer guidance on when each approach can be used and emphasises that both are valuable tools in the IPDMA field. Our examples primarily focus on IPDMA of randomised trials to examine intervention effects, but the key messages also apply to most other situations where an effect estimate is of interest (e.g., prognostic factor studies).

## 2 ONE‐STAGE APPROACH IS PREFERRED WHEN MOST STUDIES HAVE SPARSE NUMBERS OF PARTICIPANTS OR EVENTS

In a two‐stage IPDMA approach, the effect estimates derived for each study in the first stage are assumed to follow a normal sampling distribution with a known variance in the second stage.^5^, ^10^, ^11^, ^12^, ^13^ These assumptions are sensible if most studies have moderate to large sample sizes, as effect estimates derived using maximum likelihood estimation are asymptotically normally distributed, and variances can be estimated with reasonable accuracy.^13^ However, these assumptions are unreliable when most of the included studies are small (e.g., <20–30 participants in each group) or, specifically for binary, count and time‐to‐event outcomes, when most studies have few (e.g., <10) or no outcome events in one or more groups.^5^ Furthermore, effect estimates (e.g., odds ratios) in generalised linear models, such as logistic regression, are upwardly biased when outcome events are sparse,^14^, ^15^ unless a debiasing approach such as Firth's correction is used.^14^

In contrast, by analysing all the IPD together in a single model (e.g., a general or generalised linear mixed model accounting for clustering of participants within studies^5^, ^16^) the one‐stage IPDMA approach avoids making assumptions of normality and known variances of effect estimates in each study. In other words, the one‐stage approach allows a more exact likelihood specification in the statistical modelling. This also helps avoid the need for continuity corrections, often employed in the first stage of the two‐stage approach when studies have zero outcome events in one of the (treatment) groups. Therefore, a one‐stage approach is recommended in situations where most of the included studies are small in terms of the number of participants or outcome events.

For example, Box 1 presents an IPDMA of seven randomised trials with sparse outcome events in most trials. Performing a two‐stage approach (with continuity corrections of +0.5 where necessary in the first stage) gives a summary odds ratio of 1.31 (95% CI: 0.33–5.16) and a between‐trial variance estimate of zero, whilst a one‐stage approach gives a larger summary odds ratio of 1.91 (95% CI: 0.36–10.15) and a larger between‐trial variance estimate of 0.57.^17^, ^18^

BOX 1 **Example of a situation where a one‐stage IPDMA is preferred to a two‐stage approach. Case study uses data from Simmonds and Higgins,^17^ as adapted by Riley and Debray,^18^ who combine IPD from seven randomised trials examining the effect of hormone replacement therapy (HRT) on the incidence of heart disease.**

*Data*: Aggregate data derived from the IPD are shown for each trial below.

**Table:** 

Trial	No. women		No. cardiovascular events	
	Control	Treatment	Control	Treatment
1	174	701	0	5
2	14	15	1	0
3	16	15	0	1
4	20	20	1	1
5	26	29	0	1
6	84	84	3	1
7	66	68	0	3

*Methods*: We used these aggregate data to reconstruct the IPD, with a row for each participant in the dataset denoting their trial, treatment group, and outcome event. We then used maximum likelihood (ML) estimation to fit a one‐stage logistic regression model to this IPD, with the intercept stratified by trial and assuming random treatment effects. This one‐stage approach directly models the binomial likelihood, and so handles trials with zero events in one group.

To improve ML estimation of the between‐trial variance we used trial‐specific centering of the treatment group variable (i.e., 1/0 for treatment/control minus the proportion of participants in the treatment group for that trial). Confidence intervals were derived using the *t*‐distribution with six degrees of freedom.

A two‐stage approach was also applied. In the first stage, a logistic regression model was fitted using ML estimation to each trial separately, with continuity corrections of +0.5 added in trials with zero events, to obtain a treatment effect estimate (log odds ratio, ${\hat{\theta }}_{i}$) and its variance (var(${\hat{\theta }}_{i}$)) for each trial. Using these aggregate data, the second stage then fitted a standard random‐effects meta‐analysis model using REML estimation with confidence interval derived by the Hartung‐Knapp Sidik‐Jonkman approach.

*Results*: The one‐stage approach gives a summary odds ratio ($\exp\left(\hat{\theta }\right)$) of 1.91 (95% CI: 0.36 to 10.15), and between‐trial variance (${\hat{\tau }}_{\theta }^{2}$) of 0.57. The wide confidence interval and large heterogeneity suggest the findings are inconclusive and additional trials are required to investigate the association between HRT and cardiovascular disease risk. Results are very different when applying a two‐stage approach with continuity corrections, as the summary odds ratio is 1.31 (95% CI: 0.33 to 5.16) and ${\hat{\tau }}_{\theta }^{2}$ is 0.

## 3 UNLESS DATA ARE SPARSE, EVIDENCE SHOWS TWO‐STAGE AND ONE‐STAGE IPDMA RESULTS CLOSELY AGREE, PROVIDED THEY USE THE SAME MODELLING ASSUMPTIONS AND ESTIMATION METHOD

Several articles have investigated the difference between one‐stage and two‐stage IPDMA results,^6^, ^7^, ^8^, ^9^, ^10^, ^19^, ^20^, ^21^, ^22^, ^23^, ^24^, ^25^, ^26^, ^27^, ^28^ either empirically, via simulation, or theoretically. Where the estimand is a summary effect of a particular variable (e.g., a treatment effect in terms of a mean difference, odds ratio or hazard ratio), most studies conclude that one‐stage and two‐stage approaches give very similar results, except when most studies have sparse numbers of participants or events. For example, for binary outcomes, Stewart et al.^8^ conclude that ‘*one‐stage statistical analyses may not add much value to simpler two‐stage approaches*’. For time‐to‐event outcomes, Bowden et al.^9^ conclude: ‘*there appears to be only a very small gain in fitting more complex and computationally intensive one‐stage models*’. For continuous outcomes, Morris et al.^7^ concluded that ‘*the number of stages used to fit this model is irrelevant*’ as ‘*provided the same underlying model is used, inference from one‐ and two‐stage procedures is practically equivalent*’.

**FIGURE 1:**
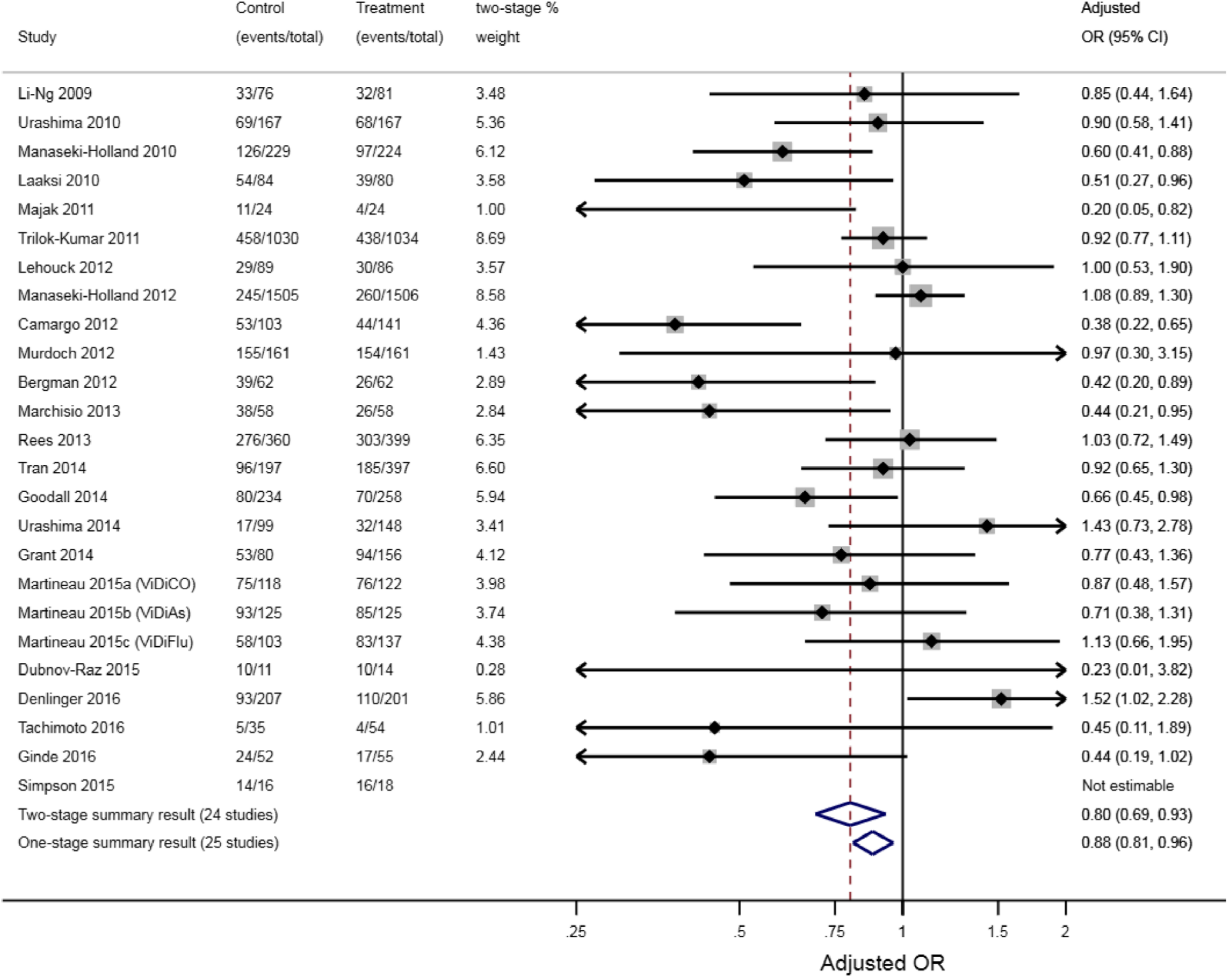
Example of a notable difference between one‐stage and two‐stage IPDMA results, as reported by Martineau et al.^31^ who synthesise IPD from 25 randomised controlled trials (10,933 participants) to examine whether Vitamin D supplementation prevented acute respiratory tract infections. Case study and figure originally shown by Riley et al.^29^ [Colour figure can be viewed at wileyonlinelibrary.com]

In practice, researchers may observe non‐negligible differences between one‐stage and two‐stage IPDMA results. Box 2 catalogues the reasons why this can happen.^29^, ^30^ Observed differences are usually due to researchers using different models, assumptions or estimation methods, rather than using one or two stages per se. For example, Figure 1 shows results from an IPDMA,^31^ with quite a large difference in the summary treatment effect from a one‐stage approach (odds ratio = 0.88, 95% CI: 0.81–0.96) and a two‐stage approach (odds ratio = 0.80, 95% CI: 0.69–0.93). Our instinct may be to conclude that this difference is due to the one‐stage approach using a model with a more exact likelihood; however, most trials are large, and events are not sparse, therefore the two‐stage approach should be a good approximation here. Instinct might also blame the exclusion of the Simpson trial from the two‐stage approach, due to an adjusted odds ratio estimate being inestimable in that trial (the authors say^31^: ‘an estimate for the effect of the intervention in the study could not be obtained in the regression model owing to small sample size’). However, the Simpson trial is very small and so should have very little contribution to the one‐stage meta‐analysis anyway.

BOX 2 **Key reasons why researchers may observe non‐negligible differences between one‐stage and two‐stage IPDMA results; adapted from Riley et al.^29^ and Burke et al.^30^**

- *More exact one‐stage likelihood versus approximate two‐stage likelihood*—when most studies are small (few participants or events), the assumed normal likelihood for the model in the second stage of the two‐stage approach can be a poor approximation.^5^
- *How clustering of participants within studies is modelled*—for example, a two‐stage approach estimates the intercept separately by study—but a one‐stage model ignores clustering entirely or assumes a random intercept.^4^
- *Specification of nuisance parameters, such as prognostic factor and adjustment terms, and residual variances*—for example, stratified by study in the two‐stage approach, but assumed common or random in a one‐stage model.^29^
- *Different estimation methods for τ* ^*2*^—for example, DerSimonian and Laird to fit the second stage of a two‐stage model and maximum likelihood estimation to fit the one‐stage model.
- *Choice of common effect or random effects for the parameter of interest (e.g., treatment effect)*.
- *Derivation of confidence intervals*—for example, the one‐stage approach may derive intervals using Wald‐based confidence intervals, but the two‐stage approach may use a *t*‐distribution.^32^
- *Accounting for correlation amongst multiple outcomes or time‐points*—for example, the one‐stage approach may synthesise all time‐points together accounting for their correlation, whilst the two‐stage approach may synthesise results for each time‐point separately in the second stage.^34^
- *Handling of within‐study and across‐study relationships*—for example, a two‐stage approach may summarise treatment‐covariate interactions based on only within‐trial information but a one‐stage model may amalgamate within‐trial and across‐trial information.^35^
- *Different studies or participants included in the one‐stage and two‐stage approaches*.
- *Differences in other modelling choices*—for example, how non‐linear trends are modelled, the set of adjustment factors included, the use of automated selection procedures, and so forth.
- *Unintentional errors*—for example, pooling odds ratios (rather than log odds ratios) in the second stage of the two‐stage approach.

Rather, the difference is due to the use of different estimation methods to fit the random‐effects models: maximum likelihood estimation for the one‐stage model and DerSimonian and Laird for the second stage model of the two‐stage approach. These lead to different estimates of between‐study variance (${\hat{\tau }}^{2}$), different trial weights in the meta‐analyses, and subsequently different summary treatment effect estimates and confidence intervals. If we rather constrain ${\hat{\tau }}^{2}$ to be the same in one‐stage and two‐stage analyses, the results are practically identical. For example, a two‐stage IPDMA constraining ${\hat{\tau }}^{2}$ to be zero gives a summary OR of 0.88 (95% CI: 0.81–0.96), identical to results from the one‐stage model (Figure 1). Hence, the decision here is more about choosing the best estimation method, rather than deciding between a one‐stage or two‐stage approach—indeed, simulation studies suggest REML estimation is preferred for either the one‐stage or two‐stage approaches assuming random treatment effects, unless outcome events are sparse in most studies.^32^, ^33^

## 4 ONE‐STAGE TO RULE THEM ALL? CLAIMS TO ALWAYS USE THE ONE‐STAGE APPROACH ARE MISLEADING

Some previous claims of superiority for the one‐stage approach are unfair. For example, Mathew and Nördstrom suggest that ‘*significant loss of precision may result from using the two‐step IPD meta‐analysis estimator*’.^26^ However, their main example of an inequality was when the intercept term was assumed common to all trials, which is of no practical interest as it breaks randomisation in each trial.^4^

Similarly, Kontopantelis conclude that ‘*a fully specified one‐stage model should be preferred, especially when investigating interactions*’ because it leads to more precise interaction estimates.^6^ However, on inspection of their simulation results, there is nothing to separate the two procedures when focusing on the overall treatment effect in the absence of an interaction. When examining treatment‐covariate interactions, the one‐stage model performs better but only because it allowed *both* within‐trial and across‐trial information to contribute (whereas the two‐stage approach only used within‐trial information). Incorporation of across‐trial information introduces aggregation bias due to trial‐level confounding and breaks the within‐trial exchangeability afforded by randomisation.^36^, ^37^ The setting investigated by Kontopantelis was the very narrow situation of no trial‐level confounding, but trial‐level confounding often occurs in practice. Further, if desired, the two‐stage approach can also be extended to combine both within‐trial and across‐trial information, by pooling the summary estimate from a meta‐analysis of within‐trial interaction estimates with the across‐trial association estimate from a meta‐regression.^35^ Thus, their general recommendation to prefer one‐stage models is not justified or fair.

Statisticians might envisage that a one‐stage model is more powerful as it estimates all parameters simultaneously. However, in situations where all parameters are estimated in every study, and nuisance parameters are stratified by study, accounting for their correlation has surprisingly little impact on the summary effect of interest.^23^ Gains only arise if stronger assumptions are made that are hard to justify, such as placing between‐study distributional assumptions on nuisance parameters.

## 5 ONE‐STAGE‐FITS‐ALL? PERCEIVED FLEXIBILITY OF ONE‐STAGE MODELS IS DECEIVING

Some researchers advocate the one‐stage approach due to its ‘increasing flexibility’ in the modelling, in terms of the assumptions or model specification. For example, one‐stage models can easily specify residual variances to be the same in every study^38^; study intercepts to be drawn from some distribution, or study baseline hazard functions to be proportional. However, the flipside to this ‘flexibility’ is that it can lead to modelling mistakes (e.g., ignoring clustering) and unjustified strong assumptions, which may produce biased or overly precise conclusions.^4^, ^30^, ^39^ Further, many bespoke one‐stage models can be replicated in a two‐stage approach anyway,^40^ for example by extending to a multivariate framework in the second stage to place distributional assumptions on nuisance parameters, such as residual variances.^41^, ^42^

Therefore, the promise of one‐stage ‘flexibility’ is often more about convenience than a scientific advantage. It is even deceiving, as the two‐stage approach could also be portrayed as more flexible than the one‐stage approach. For example, when synthesising studies with different and complex designs (e.g., cluster trials, parallel‐group trials), the first stage of the two‐stage approach can easily tailor models to address the design of study,^43^ whereas this is more challenging in a one‐stage approach (though possible^34^, ^44^, ^45^). A two‐stage approach is also more practical when the IPD from all studies cannot be harmonised altogether (e.g., if data sharing agreements for some studies only allow IPD to be accessed remotely at the host institution), or when needing to include aggregate data from studies not providing IPD.^1^ Visualisation of a two‐stage meta‐analysis is also easier, for example via a forest plot containing study‐specific estimates, percentage study weights and summary results.

Finally, the two approaches do not make any different default assumptions about missing data: complete case analysis is valid when missing outcomes and missing covariates are missing depending only on the values of (other) covariates included in the model. If the approach to handling missing data needs to differ in each study, this may be more convenient to handle in the first stage of the two‐stage approach via study‐specific (imputation) models. However, if there are systematically missing covariates, a one‐stage model for imputation may be preferable to allow borrowing of information across studies.^46^ Regardless of how missing data are handled (e.g., whether missing values are multiply imputed based on a one‐stage model or study‐specific models), in principle either two‐stage or one‐stage IPD meta‐analysis models could still be used for the main analysis if they are congenial with the imputation model.

## 6 DISCUSSION AND RECOMMENDATIONS

So, two‐stage or not two‐stage? If most studies in the IPDMA are small (few participants or events), a one‐stage approach is recommended. In other situations, researchers can choose either approach, carefully following best practice.^1^ Although one‐stage models are a suitable choice, they require much care to specify correctly (see example code at https://www.ipdma.co.uk/one-stage-ipd-ma) and deal with complexities, such as centering covariates,^32^ and separating within‐study and across‐study relationships.^35^ For this reason, unless data are sparse, the two‐stage approach will often suffice, with dedicated software options either for both stages (ipdmetan^47^) or just the second stage (e.g., metan,^48^ and metafor^49^). Where feasible, it can be helpful and transparent to do both one‐stage and two‐stage analyses, and report both. If their results are appreciably different, it is important to identify and understand why, guided by the reasons listed in Box 2 and related guidance.^29^

## AUTHOR CONTRIBUTIONS

**Richard D. Riley:** Conceptualization; investigation; methodology; writing – original draft; writing – review and editing; software; formal analysis; data curation; supervision; project administration. **Joie Ensor:** Writing – review and editing; methodology; investigation; project administration. **Miriam Hattle:** Writing – review and editing; methodology. **Katerina Papadimitropoulou:** Writing – review and editing; methodology. **Tim P. Morris:** Methodology; writing – review and editing.

## FUNDING INFORMATION

Richard D. Riley and Joie Ensor were supported by an MRC‐NIHR Better Methods Better Research grant (reference: MR/V038168/1), and the NIHR Birmingham Biomedical Research Centre at the University Hospitals Birmingham NHS Foundation Trust and the University of Birmingham. Tim P. Morris is funded by the UKRI Medical Research Council (grants MC_UU_00004/06 and MC_UU_00004/07). The views expressed are those of the author(s) and not necessarily those of the NHS, the NIHR or the Department of Health and Social Care.

## CONFLICT OF INTEREST STATEMENT

Richard Riley is the lead Editor on the book ‘Individual Participant Data Meta‐Analysis: A Handbook for Healthcare Research’ for which he receives royalties.

## Data Availability

Data sharing not applicable to this article as no datasets with individual participant data were generated or analysed during the current study. The aggregate data for the trials of the two main examples is already provided in Figure 1 and Box 1.
